# A Pathfinding Algorithm for Lowering Infection Exposure of Healthcare Personnel Working in Makeshift Hospitals

**DOI:** 10.3390/healthcare10020344

**Published:** 2022-02-10

**Authors:** Braxton Rolle, Ravi Kiran, Jeremy Straub

**Affiliations:** 1 Department of Computer Science, North Dakota State University, Fargo, ND 58105, USA; braxton.rolle@ndsu.edu (B.R.); jeremy.straub@ndsu.edu (J.S.); 2 Department of Civil & Environmental Engineering, North Dakota State University, Fargo, ND 58105, USA

**Keywords:** field hospitals, pathfinding AI, air-borne diseases, COVID-19, front-line workers

## Abstract

Due to the recent COVID-19 outbreak, makeshift (MS) hospitals have become an important feature in healthcare systems worldwide. Healthcare personnel (HCP) need to be able to navigate quickly, effectively, and safely to help patients, while still maintaining their own well-being. In this study, a pathfinding algorithm to help HCP navigate through a hospital safely and effectively is developed and verified. Tests are run using a discretized 2D grid as a representation of an MS hospital plan, and total distance traveled and total exposure to disease are measured. The influence of the size of the 2D grid units, the shape of these units, and degrees of freedom in the potential movement of the HCP are investigated. The algorithms developed are designed to be used in MS hospitals where airborne illness is prevalent and could greatly reduce the risk of illness in HCP. In this study, it was found that the quantum-based algorithm would generate paths that accrued 50–66% less total disease quantum than the shortest path algorithm with also about a 33–50% increase in total distance traveled. It was also found that the mixed path algorithm-generated paths accrued 33–50% less quantum, but only increased total distance traveled by 10–20%.

## 1. Introduction

The COVID-19 pandemic brought many challenges to healthcare systems worldwide and caused over 4 million deaths, which are rising still [1]. Many hospitals were overrun, and makeshift (MS) hospitals started popping up wherever they were needed, but they did not have the same ventilation and protection as a normal hospital would. These MS hospitals were only regulated to have exhaust air volume of 150 m^3^ per hour per person, whereas the guideline for infection control by the WHO is 288 m^3^ per hour per person [2]. On top of this, after the outbreak started, not much was known about COVID-19, which resulted in a lack of proper protection for healthcare workers. During the initial stages of the pandemic, in Wuhan, China, the infection rate among healthcare workers was anywhere from 3.5% to 29% throughout various hospitals [3]. This infection rate is extremely high and eventually would drop as PPE regulations were put into place and healthcare workers were supplied with proper protection and training. However, healthcare workers were still one of the highest risk groups in the pandemic. The most reported reasons for this were the lack of PPE, followed by work overload and lack of proper hygiene or inadequate usage of PPE. By April 2020, 10.7% of total cases in Italy were healthcare workers, and almost 14% of total cases in Spain were healthcare workers [4]. At one point in the United States, healthcare workers made up as much as 19% of total patients admitted for COVID-19 [5]. Of course, after PPE regulations were fully fleshed out and all workers were supplied with the correct protection, the rate of infection for healthcare workers dropped, but many still faced mental health struggles. One study surveyed over 8800 hospital workers in Chongqing and found that 30.7% had symptoms of depression, 20.7% had symptoms of anxiety, and 6.5% thought about self-harm and/or suicide [6]. Another study surveyed over 5000 workers in Spain during the first wave of the outbreak, May to July 2020, and found that 8.4% experienced suicidal thoughts and/or behaviors. The biggest factors given were the lack of workers, supervision, coordination, or communication while at work and financial stress [7].

Most countries used MS hospitals to combat the spread of COVID-19, especially at the start of the outbreak. In China, Fangcang hospitals were deployed. These ‘hospitals’ were basically just large public venues temporarily converted into healthcare facilities [8]. These facilities provided care to people with mild to moderate symptoms and isolated them from their communities but did not contain many if any intensive care capabilities and, instead, were built to help established hospitals contain the overflow of patients. These hospitals were extremely effective at accomplishing what they were designed to do, and according to one study, if the Fangcang hospitals opened only one day later, there would have been about a 14,581% increase in total cases, a 27,903% increase in deaths, and would have lasted over 100 days longer [9]. By May 2020 in the United States, over 660 million USD was spent renovating convention halls, university buildings, and abandoned warehouses into general care overflow hospitals [10]. Most of these MS hospitals were never used due to poor planning, but one had 1000 total patients over the course of a few weeks. Some countries focused on providing more intensive care rooms. In France, an MS hospital was constructed with 30 ICU beds to provide extra care alongside the Émile-Muller hospital in Mulhouse [11]. This was the first of its kind and was fully equipped with all needed critical care equipment. It was connected to the Émile-Muller hospital’s computer system for access to medical records and for cases of patient transfer. It also was divided into three separate zones, each requiring varying levels of PPE to ensure proper precautions were taken throughout the MS hospital. It was open from 24 March 2020 to 17 April 2020, and held 46 critical care patients over this time.

Disease outbreaks, like any other disaster, can happen at any time, and if healthcare systems are unprepared to handle it, they can quickly turn into a global crisis. Many hospitals were not adequately prepared for COVID-19, which is why it was able to spread so quickly and put so much strain on global healthcare systems. A study carried out in Nigeria found that only 15% of the hospitals they surveyed would have been adequately prepared for COVID-19 [12]. Another study found that hospitals on average in the United States only had around 0.8 beds per 1000 people on a normal day, meaning there were very few beds for COVID-19 patients [13]. After many countries realized they were not prepared to handle the oncoming global epidemic, they started to find ways to estimate the number of COVID-19 cases using various types of modeling so that they could improve their healthcare systems and be ready for the new influx of sick patients. Common models used in the current research field include SIR and SEIR models, which are able to closely predict the number of cases of an infectious disease over a period of time. These models can be used to allow governments to predict if, or when, a disease will surpass a threshold of infected people in which action must be taken to stop further spread of the disease [14]. One study implemented a stochastic forecast model to predict the number of COVID-19 cases in both the first and second waves in Sri Lanka. This model was found to have potential benefits over the others in closely predicting how many people will become infected over a period of time [15]. Other researchers used data mining to study environmental and meteorological variables and determine their correlation with the number of COVID-19 cases in three cities in Brazil. The model they developed was successful in predicting the number of cases and deaths in the cities they studied [16]. There was plenty of work carried out on predicting the number of cases. However, we were unable to find studies that focused on helping HCP stay safe while maneuvering hospitals.

Pathfinding algorithms have been around for several decades. Dijkstra’s algorithm was conceived in the late 1950s [17]. However, new implementations are still being found for them. Common applications of these algorithms include video games [18] and GPS [19]. In more recent years, pathfinding has expanded into the fields of robotics [20] as well as emergency response services [21]. Dijkstra’s algorithm finds the shortest path between two nodes on a graph by searching all the surrounding nodes, with the ones closest to the start being prioritized. This approach is highly inefficient as the further away the target is, the longer the search is going to take, and for implementations where the calculations need to be performed extremely quickly, Dijkstra’s algorithm is not usually the best choice for pathfinding. This is where the A* algorithm improves greatly. A* was developed in 1968 and uses a heuristic function to speed up the searching process [22]. Both of these algorithms will generate the shortest paths. However, A* will never run slower than Dijkstra’s due to its informed method of searching. This makes the A* algorithm very common in implementations for video games, which require the pathfinding to occur in real-time, or very close, in order to create an enjoyable experience for the player [23].

Healthcare workers are the backbone of the healthcare system and need to be kept as safe as possible if an outbreak were to occur so that they can effectively help other patients without furthering the spread of the disease. Currently, hospitals are working on making things safer for HCP, but very few studies, if any, have been published on helping HCP navigate MS hospitals safely. When dealing with an airborne illness, staying distanced from infective people is incredibly important, especially in areas where personal protective equipment (PPE) is in low supply. This study focuses on keeping workers away from infective patients as much as possible while they are moving through the hospital, which is an area that we could not find much, if any, research being conducted.

This study aims to provide a pathfinding algorithm that would make the workplace safer for healthcare workers, specifically in MS hospitals, and would better prepare healthcare systems for an airborne epidemic or pandemic if one were to happen again. The goal of this study is to reduce the risk of infection in healthcare workers in MS hospitals dealing with airborne diseases. The objective of this study is to implement a pathfinding algorithm into a mock MS hospital to find the safest, fastest, and most effective paths for the healthcare workers to traverse from one position to another and limit the amount of disease quantum they accrue. The shortest path between two points in a hospital is not always the safest and the safest path may not be viable as it could be too long or tortuous. An algorithm that produces a path with lower infection exposure to the HCP without unreasonably increasing the traversable length of the path is the important contribution of this work.

## 2. Overview

This study begins with a validation and explanation of a commonly used pathfinding algorithm, as well as a demonstration of how it works in the Methodology section. This algorithm is then applied to work in a hypothetical makeshift hospital in the Shortest Path Algorithm for MS Hospitals section. The layout of this hospital is discussed, followed by the rules of the tests and how they are conducted. A base test consisting of a shortest path algorithm and the hypothetical hospital is discussed.

The effects of the grid refinement are then tested, meaning the increasing or decreasing of units on the grid while maintaining the same overall grid size. The degrees of freedom (DoF), the number of units the algorithm can move to from the current unit, of the algorithm’s movement is then discussed, followed by testing of its impact on the algorithm’s function. A lower DoF is tested on the algorithm. This is then used in a test on total occupancy percentages in a hospital. Various occupancy percentages and hospital sizes are used.

**Figure 1:**
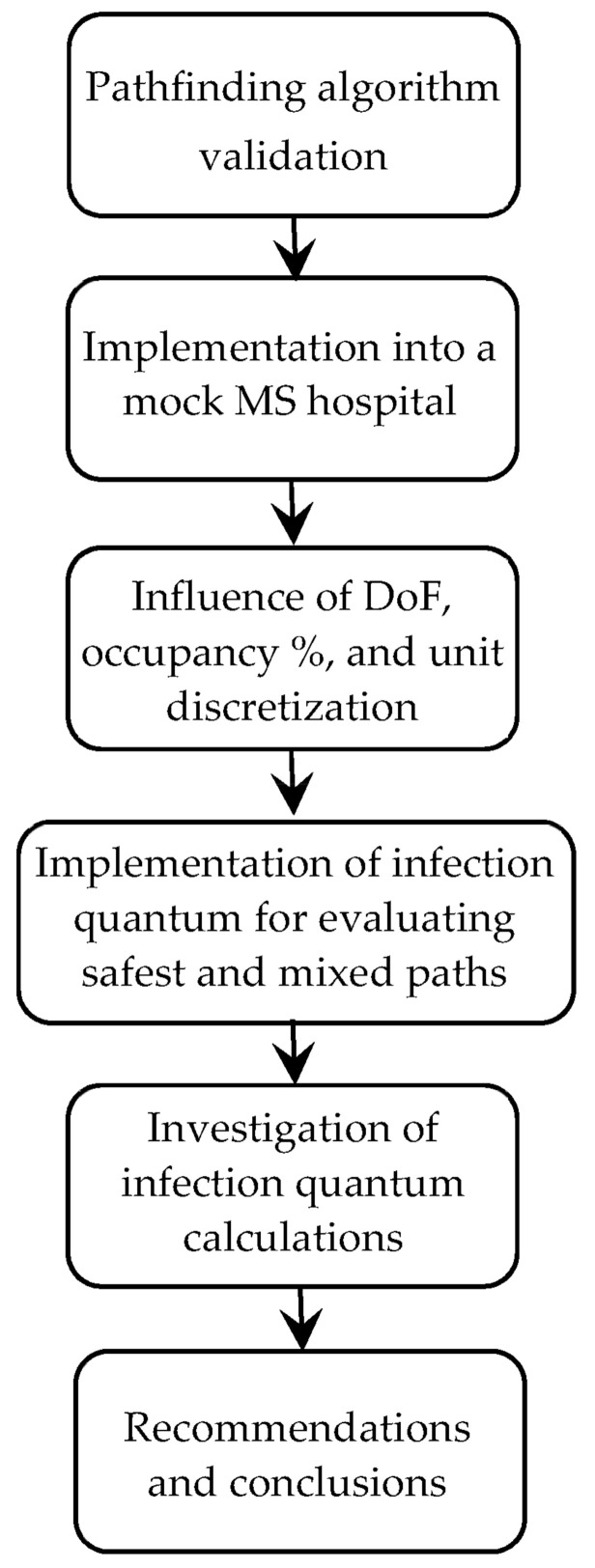
Overall flowchart of the paper.

After this, the safest and mixed path algorithms are introduced in the Safest and Mixed Paths section. These tests use a dummy airborne infection model to simulate COVID-19 exposure to workers. The ‘safest’ path algorithm follows only quantum exposure and aims to avoid the most exposure possible with no regard to how far the worker must travel, whereas the mixed path algorithm aims to balance both the distance traveled while still minimizing infection exposure. These two algorithms are used in the same tests as before and the results are compared to those from the shortest path algorithm. To finish this study, the variables used in the infection exposure calculation were modified to view their effect on the algorithm’s function in the Investigation of Infection Exposure Calculations section. A flowchart of the paper and its primary sections is shown below in Figure 1.

## 3. Methodology

All the tests conducted in this study used a 2D grid similar to a graph as a platform for the pathfinding AI to work. The origin (0, 0) was in the bottom left of the grid. Moving right increased the x-value and moving up increased the y-value. The grid units used had various shapes and sizes for each individual point on the grid, but the first tests used squares as a base unit. To begin with, each unit could only be open or closed. Open units could be traversed by the algorithm and closed units could not. The starting and ending units had to be open for the algorithm to function properly.

### 3.1. Shortest Path Algorithm and Demonstration

The shortest path algorithm used in this study is a typical algorithm used in pathfinding known as A*, which considers both the distance needed to travel to reach the target and the distance traveled from the starting point when calculating the path [22]. The q-based and mixed path pathfinding algorithms used later in this study use this as a basis but adapt it to fit the needs of a hospital and airborne infection exposure, rather than creating a new algorithm from scratch. It is used to navigate the HCP through a hypothetical MS hospital and has three parameters (F, G, and H), which are used to find the shortest possible path between any two positions in the generated grid. G is the calculated Euclidean distance between the center of the starting position and the current position, which is given as

(1) $$G=\sqrt{{\left(x_{i}-x_{c}\right)}^{2}+{\left(y_{i}-y_{c}\right)}^{2}}$$

where $\left(x_{i},y_{i}\right)$ are the coordinates of the center of the initial position and $\left(x_{c},y_{c}\right)$ are the coordinates of the center of the current position. On the other hand, H is the Euclidean distance between the center of the current location to the center of target position and is given as

(2) $$H=\sqrt{{\left(x_{c}-x_{t}\right)}^{2}+{\left(y_{c}-y_{t}\right)}^{2}}$$

where $\left(x_{t},y_{t}\right)$ are the coordinates of the center of the target position. The parameter F is given as

(3) F=G+H

The F value is the primary value used in calculating the shortest possible path and is used as the value to determine which units to check while searching. The algorithm starts by searching the units surrounding the starting position, calculating their respective *F*, *G*, and *H* values, and selecting the position with the smallest F value. If two *F* values are the same, the position with the smaller *G* value will be selected.

**Figure 2:**
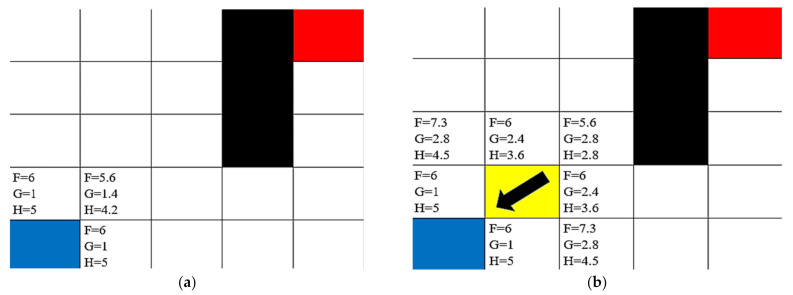
Demonstration of pathfinding algorithm with F, G, and H values labeled at (**a**) the starting position, and (**b**) the first step (blue square—starting position, black square—untraversable; red square—target square; and yellow square—path).

**Figure 3:**
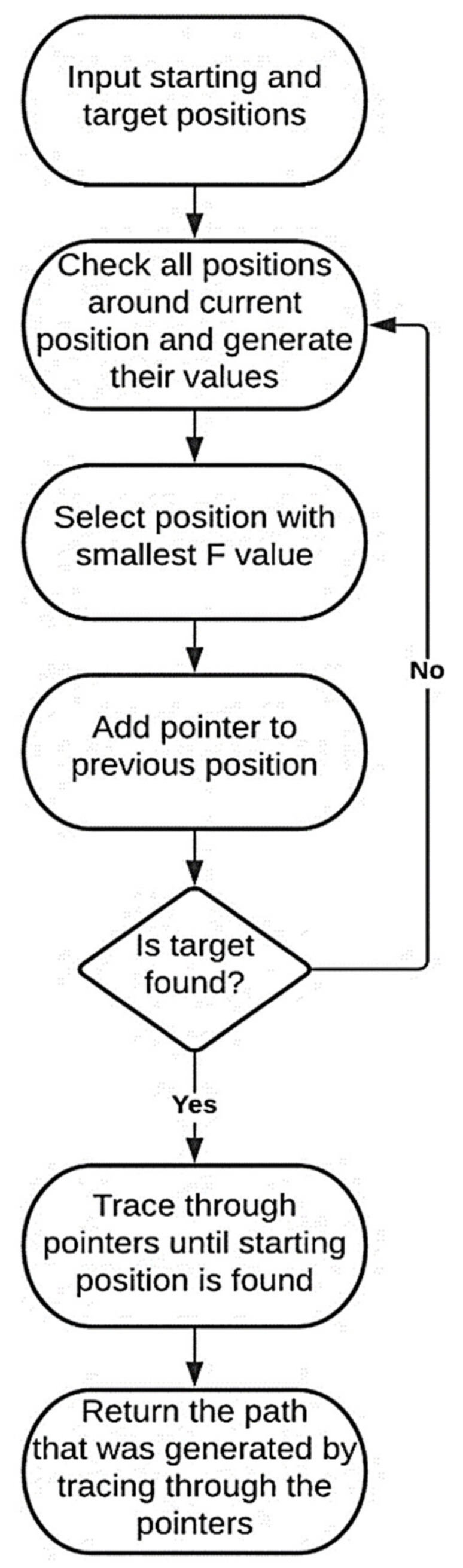
Flowchart describing the shortest path A* algorithm.

In Figure 2, the blue square is the starting position, and the red square is the target position. The black squares are not traversable, so the F, G, and H values are not calculated. The yellow square was selected as the “best” position to traverse as it had the lowest F value when compared to all the feasible squares. When a position is checked and selected, the position previously checked by the algorithm is stored as a pointer. This is used when the path is generated later and allows the program to just trace backward through each square following the pointer variable and store the square’s position. The positions around the yellow squares are then checked and, once again, the lowest F value is selected. This process is repeated until the target position is found and selected. Once the target is selected, the algorithm traces through the pointers of each position until the starting position is found and then returns the path that was just generated. A flow chart of this process is provided in Figure 3.

### 3.2. Shortest Path Algorithm Validation

The path generation algorithm used in this study was tested on its ability to find the shortest possible path in a 2D grid between any two units. This was tested using a 100′ × 100′ plan made up of a grid of 100 units, each 10′ × 10′. On this grid, the starting unit was selected, and the center of it had the coordinates (0, 5). The target position was randomly selected anywhere on the grid except for directly on top of the starting position. After this, a set number of squares were set to close, which made them untraversable. These closed squares were randomly selected on the grid anywhere except on top of the starting and target squares. This setup was tested with 10% and 20% total squares blocked.

**Figure 4:**
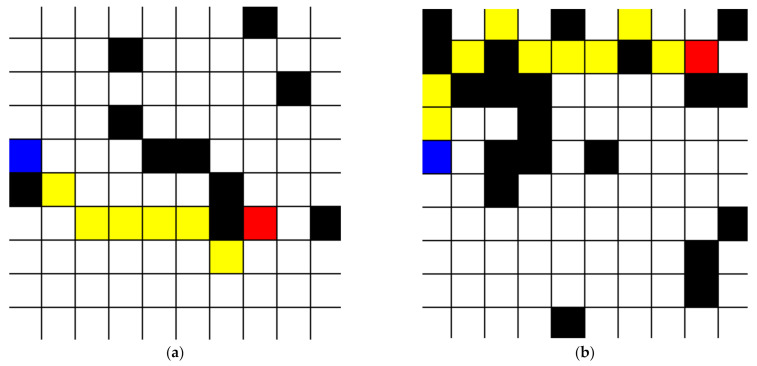
(**a**) Validation of pathfinding algorithm with 10% total area blocked, and (**b**) 20% total area blocked (blue square—starting position; black square—untraversable; red square—target square; and yellow square—path).

The results, shown in Figure 4a,b below, demonstrate the algorithm’s ability to create the shortest possible path from the starting point to the target point. Both the 10% and 20% blocked square tests were run 15 times and every time the shortest possible path was taken. The path was verified as the shortest possible manually each time by measuring the distance that the algorithm’s path took and then checking it with the distance of paths that were close in distance or potentially shorter. The only complication was when the target or starting square was completely surrounded by blocked squares, but this happened only once and demonstrates a problem with the test rather than the algorithm itself. These results show that this algorithm can reliably be used within the bounds of this study as the shortest possible path algorithm.

## 4. Shortest Path Algorithm for MS Hospitals

For this study, a hypothetical MS hospital with 200′ × 200′ plan dimensions was chosen, and its plan was modeled using a 2D grid. The MS hospital is assumed to have 16 total wards where patients can be housed with entrances in the northwest. We assumed the patient occupancy rates to be between 25% and 50% at any given time. The control room (CR) where the HCP and hospital supplies are housed is located in the central west of the hospital and will be the starting point for the HCP. These rules were incorporated into the layout used by the algorithm in order to simulate how effective the shortest path algorithm was in a realistic setting.

The code selects 4–8 different patient wards of the MS hospital specified above and simulates three HCP agents seeking out these patients and assisting them by traveling the shortest distance possible. The number of three HCP was chosen for this study because it would give the MS hospital between a 1:1.33 and 1:2.66 nurse-to-patient ratio depending on how many patients are present, which is as close as it can get to the benchmark of a 1:2 nurse-to-patient ratio that is aimed for in Intensive Care Unit rooms [24]. All three HCP start in the same control room at the same time, and each is given a target patient that they must help.

The movement of the HCP is governed by a set of 5 rules: (1) they are not allowed to traverse through the control room, as in moving through it while searching for or moving towards a patient; (2) they cannot travel through patient rooms or empty wards; (3) they can only enter patient rooms from the top northwest corner; (4) they cannot be in the same position on the grid as another HCP at the same point in time; and (5) their first step must always be a unit that connects to the southwest corner of the CR.

To begin, patients are assigned to the HCP based on how close the patient is to the CR in Euclidean distance. The first HCP (green) is given the closest patient, the second HCP (pink) is given the second closest, and the third HCP (yellow) is given the third closest patient. Once a patient has been assisted, they will no longer be a possible selection for any of the HCP. After an HCP has completed their trip, they are then assigned the next closest patient to their current position until there are no more patients. The order is always the same, so if there are 4 total patients, the green HCP would always be the one to help the farthest patient, regardless of the distance to the others. This process is repeated until there are no more patients who need assistance, at which point the HCP returns to the CR.

**Figure 5:**
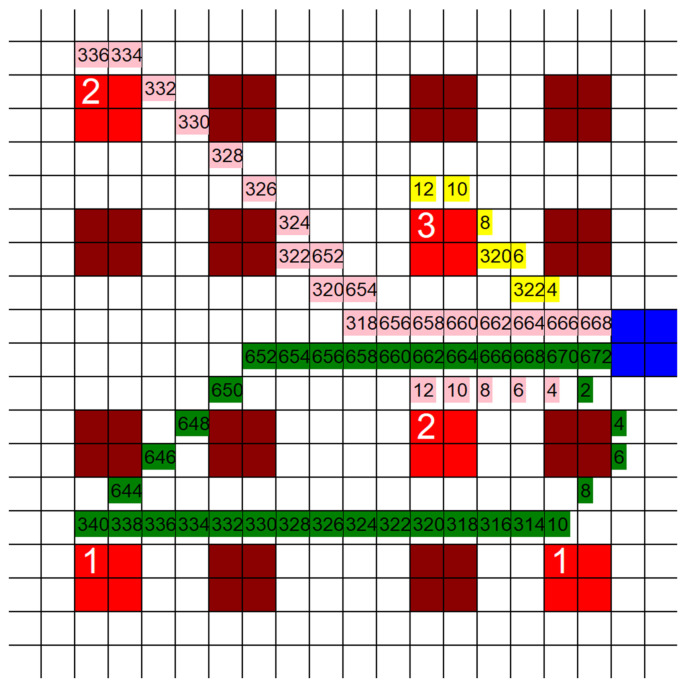
Example of a hypothetical MS hospital being used as a grid for traversal by the shortest path pathfinding algorithm. (20 unit × 20 unit grid size, 5 infective patients, 3 HCP, time in seconds in the grid).

Figure 5 is an example of what is generated when the code is run. The dark red blocks of squares are empty wards, the blue block of squares is the control room, and the bright red blocks of squares are the patient rooms. Each square represents a 10′ × 10′ area. The preferred walking speed of most people is around 1.42 m/s (4.66 ft/s) [25]. This was rounded to 5 ft/s when used in this study to account for the frantic and high-paced work environment the HCP are in. This means that each square takes the HCP about 2 s to travel through. Once the HCP reach the first ward, they take 5 min to help the patient before seeking out their next target. This amount of time was arbitrarily chosen; however, as long as the time for each patient is the same, this amount of time could be any number and it would not affect the outcome. Each color represents a different HCP and the path they took to reach each patient they were assigned. The numbers on these colors represent the time that each HCP would have been in that square in seconds. For example, if the square has the number “20”, then that means that HCP would have been at that position in the hospital 20 s after leaving the CR. The numbers that represent time are only shown to allow us to see the exact direction the HCP would have traveled. The time was only used to make sure that two HCP were not in the same position at the same time, and was not used for any actual calculations. The numbers on the bright red patient blocks of squares represent which HCP helped that patient. These basic tests yielded useful results, but it was felt that these tests needed to be expanded on, and different elements of them needed to be investigated further.

### 4.1. Effect of Grid Refinement

**Figure 6:**
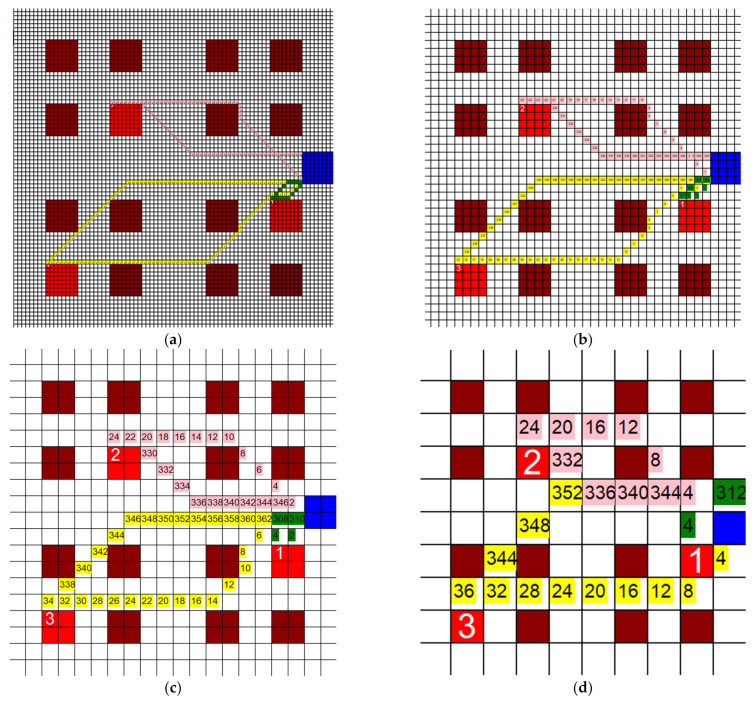
Paths taken by HCP when (**a**) 80 units × 80 units, (**b**) 40 units × 40 units, (**c**) 20 units × 20 units, and (**d**) 10 units × 10 units grids are used (3 HCP, 3 infective patients, 200′ × 200′).

**Figure 7:**
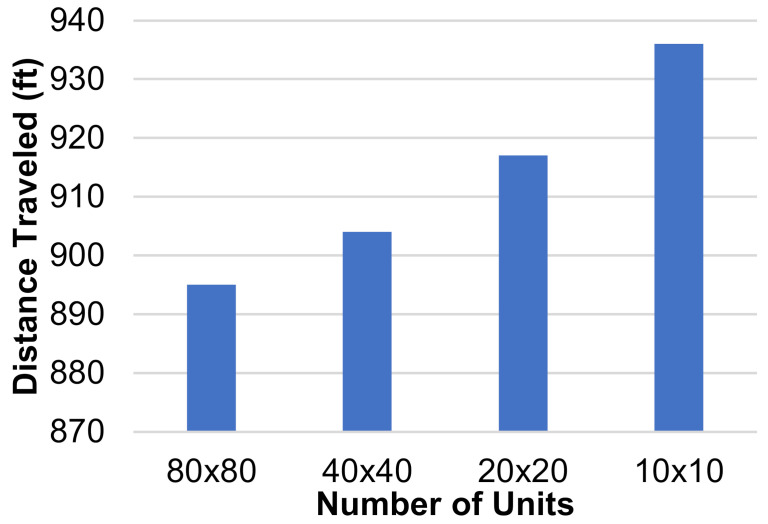
Distance traveled (ft) vs. number of grid units for a square-based grid.

The dimensions of the individual squares in the grid were altered to find the influence they had on the total distance traveled by each HCP. Three new grids were generated, two of them with a finer mesh and one of them with a coarser mesh. The coarser grid’s dimensions were 10 units × 10 units, each unit being 20′ × 20′, and the finer grid’s dimensions were 40 units × 40 units, each unit being 5′ × 5′. The third new grid had dimensions of 80 units × 80 units, where each unit represented 2.5′ × 2.5′. The layout and positioning of the patient rooms and the CR were kept the same throughout all grids. This was carried out so that the tests would be comparable. If the grids are vastly different, then the results do not give us useful information for this study, so the grids must be kept with the same relative layout as they are sized up or down. An example of one test is shown on each grid in Figure 6a–d. Three separate tests were run on each of these grids and the total distance traveled by all three agents was recorded. Each test had different active patient rooms, but between different grids, these stayed the same. The results of these tests are shown in Figure 7.

These results display that creating a finer mesh will decrease the total distance traveled by the paths generated. The distance decreases as more units are added because the algorithm has more options to generate each path. Following this trend, it can be assumed that refining the mesh further would continue to lower the total distance traveled. However, the more refined the grid becomes, the smaller the difference between these distances would be. The smallest size of the units was left at 2.5′ × 2.5′ for one main reason. Following the trend of reducing or increasing the size of the units by dividing both dimensions by 2, the next step, 1.25′ × 1.25′, would not be able to contain an adult human, so there would be no point in using that while generating paths for humans to follow.

After adjusting the size of the units in the grid/mesh, the shape of these units was adjusted to see the effect that more/fewer degrees of freedom (permissible directions of motion) while moving would have. Currently, the discretization units used were in the shape of squares and allowed for eight degrees of freedom: one for each of the cardinal directions, and then one for each diagonal between.

### 4.2. Degrees of Freedom

Degrees of freedom (DoF), when used in this study, refers to the number of possible directions that the algorithm can take from each position, assuming all surrounding positions are open. The higher this number is, the more potential moves that the algorithm can make. For example, 10 degrees of freedom would mean that the algorithm could search and move in 10 different positions from its current position.

### 4.3. Change in Discretization Unit Shape but with Same DoF

**Figure 8:**
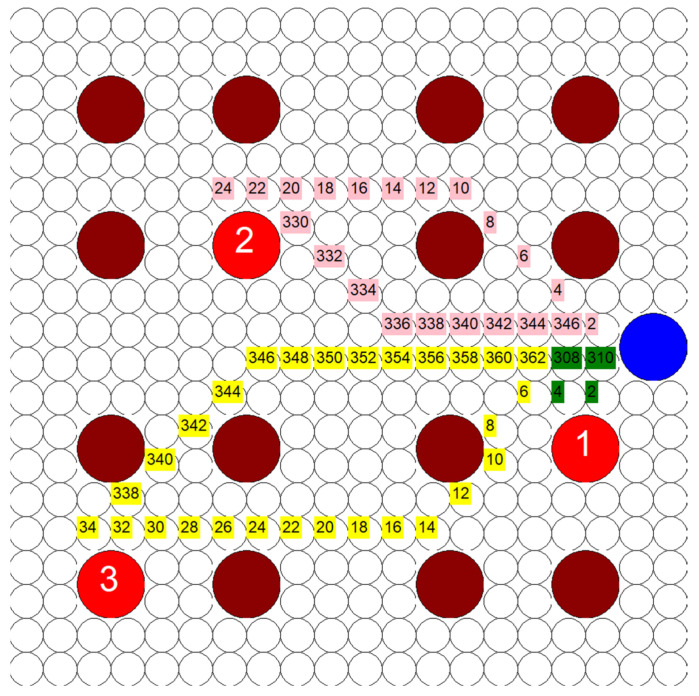
Example of a test run using circles as the fundamental discretization unit (3 HCP, 3 infective patients, 200′ × 200′ hospital layout, 20 units × 20 units).

**Figure 9:**
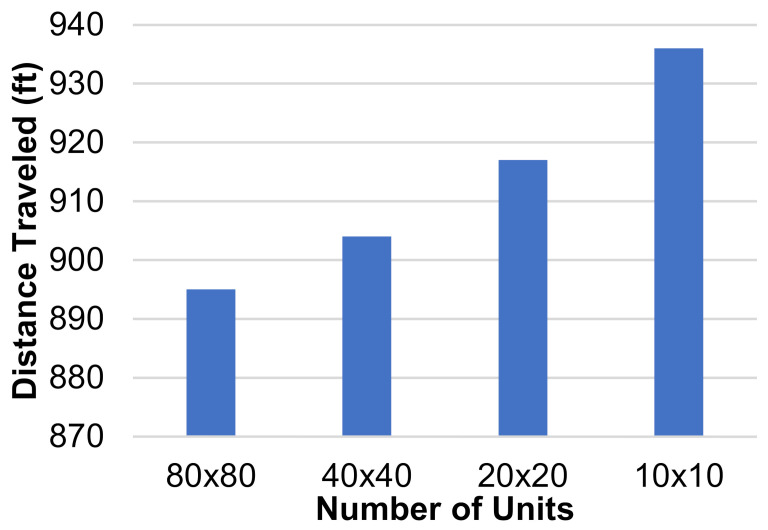
Distance traveled (ft) vs. number of grid units for a circle-based grid.

First, uniform circles were introduced as the fundamental discretization unit instead of squares. This test, like the ones prior, still used a classical A* algorithm that was adapted for use in the mock MS hospital. This did not change the outcome of how the path was generated because there was still eight degrees of freedom to move in. The only change from before was the visual design of the grid units and nothing else. An example of how this looked is shown in Figure 8 using the same test displayed in Figure 6c, and it can be seen that the paths taken by the algorithm are the exact same. Each HCP is in the same position at the same time for every position in every path for both tests. The results for the circle-based grid are shown in Figure 9.

### 4.4. Influence of Lower DoF

The second test carried out on the influence of DoF in this study used hexagons as the fundamental discretization unit rather than circles or squares. The hexagons used had a short diagonal of ten feet, and an apothem of five feet. The apothem is the distance from a flat side of the hexagon to the midpoint, and the short diagonal is the distance between two flat sides. This change in the fundamental discretization unit changes how the algorithm functions. It now uses three separate variables from before.

The E value represents the estimated distance from the current hexagon to the target hexagon. It is calculated using a modified Manhattan distance formula where only the highest difference of values is used, which is given as

(4) $$E=\mathrm{Max}\left(\left|y_{t}-y_{c}\right|,\;\left|x_{t}-x_{c}\right|\right)\times l^{*}$$

where $\left(x_{t},y_{t}\right)$ are the coordinates of the target hexagon, $\left(x_{c},y_{c}\right)$ are the coordinates of the current hexagon, and $\mathrm{Max}\left(*\right)$ returns the maximum value and $\left|*\right|$ returns the absolute value of the input. The value is then multiplied by $l^{*}=10^{\prime }$, which is the characteristic length in feet.

The T value was a representation of how far the algorithm has traveled to reach the current hexagon. No matter which direction the agent would move from each hexagon, the distance would always be $l^{*}=10^{\prime }$ more than before. T was used while searching in the algorithm, so the value was not removed completely, although its value is infrequently used. The calculation of the T value is given as

(5) $$T_{n}=T_{c}+l^{*}$$

where $T_{n}$ is the value of T for the new position being checked, and $T_{c}$ is the value of T for the current position.

The C value performs the same function as the F value used previously, except using the T and E variables. Its calculation is given as

(6) C=T+E

**Figure 10:**
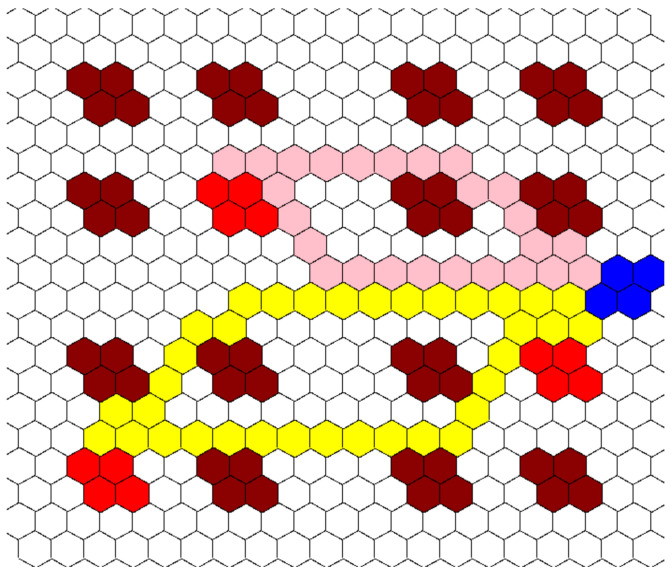
Example of algorithm working through a hexagonal grid, same color coding as in prior tests and examples (3 HCP, 3 infective patients, 200′ × 200′ hospital layout, 20 units × 20 units).

The change in the discretization unit effectively limited the DoF that the algorithm was given to calculate the path between points. Uniform circles and squares both have eight DoF that were potentially traversable from each point. However, hexagons only have six. An example of the algorithm traversing through this hexagonal grid is shown in Figure 10. This is the same test patient layout used in both Figure 6c and Figure 8.

**Figure 11:**
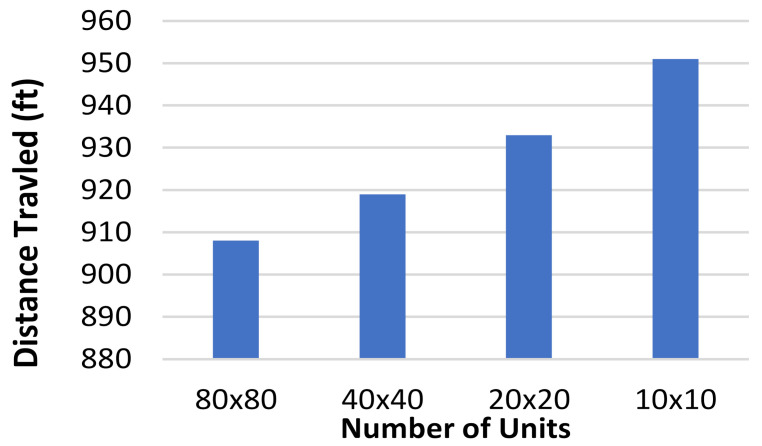
Distance traveled (ft) vs. number of grid units for a hexagon-based grid.

To further test the effects of grid refinement, three other grid unit sizes were used along with the one shown in Figure 10, and they follow the same size changes that were used before being 10 × 10, 40 × 40, and 80 × 80 grids. These grids used hexagons with a short diagonal of 20′, 5′, and 2.5′, respectively. As the unit size changed, the value added to the *G* value mirrored the change to keep accurate track of distance traveled. The total grid size was still maintained at 200′ × 200′; only the unit size was changed. The CR and patient rooms were also held to the same relative size. These grids used the same three tests that the other grid refinement tests used, and the total distance traveled by each agent was calculated. The results are shown in Figure 11.

### 4.5. Further Restricted Movement

To continue the trend of restricting DoF even further and to further test the results found in the previous section, the next tests used a square-based grid again. However, the DoF would be restricted to only four this time instead of eight. The algorithm would only be able to search and move in the four cardinal directions. Once again, this affects how the algorithm functions and the variables that must be calculated. The T value from the previous section is reused here, but two more variables are introduced.

The J value once again represents the estimated distance from the current square to the target square, and it was calculated using the Manhattan distance formula given as

(7) $$J=\left(\left|y_{t}-y_{c}\right|+\left|x_{t}-x_{c}\right|\right)\times l^{*}$$

This formula uses the same values as the hexagonal E value calculation, and the only major difference is instead of finding the max value between the two, it adds them together.

The M value functions the same as the F and C values, just with different variables, and is given as

(8) M=J+T

**Figure 12:**
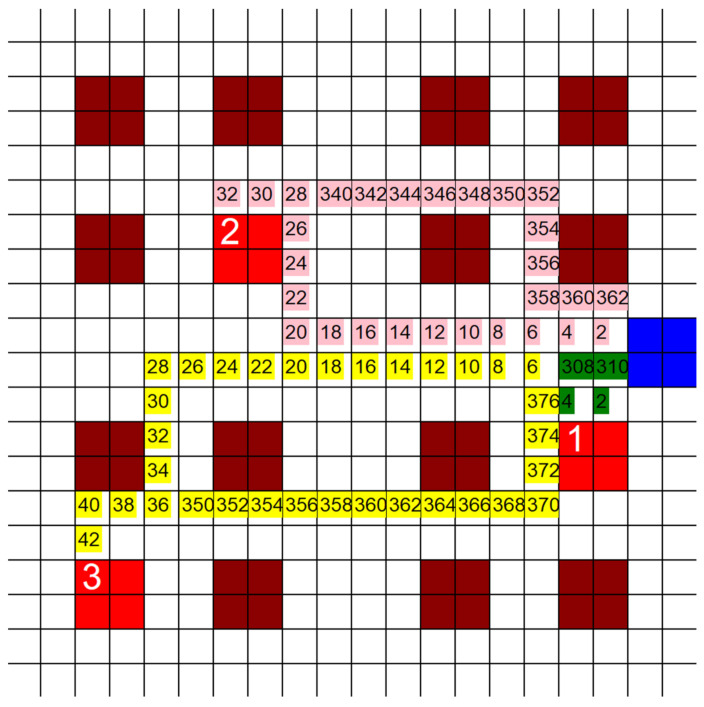
Example of algorithm working through a grid using four degrees of freedom for movement (3 HCP, 3 infective patients, 200′ × 200′ hospital layout, 20 units × 20 units).

**Figure 13:**
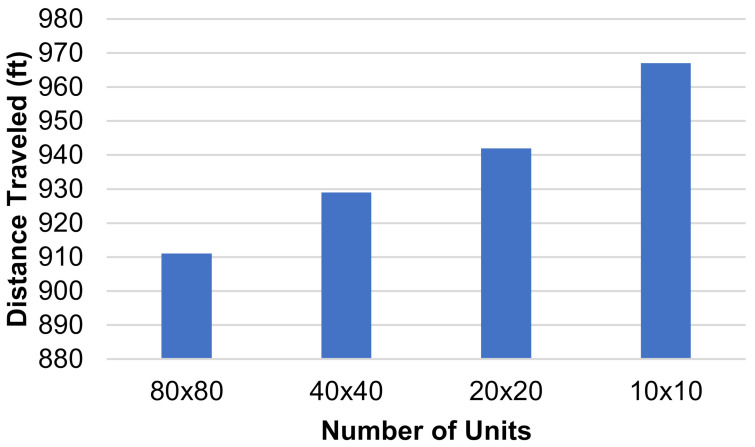
Distance traveled (ft) vs. number of grid units for a square-based grid with restricted movement.

An example of one of these tests being run is shown below in Figure 12 and uses the same layout as Figure 6c, Figure 8 and Figure 10. The results of these tests are shown in Figure 13.

**Figure 14:**
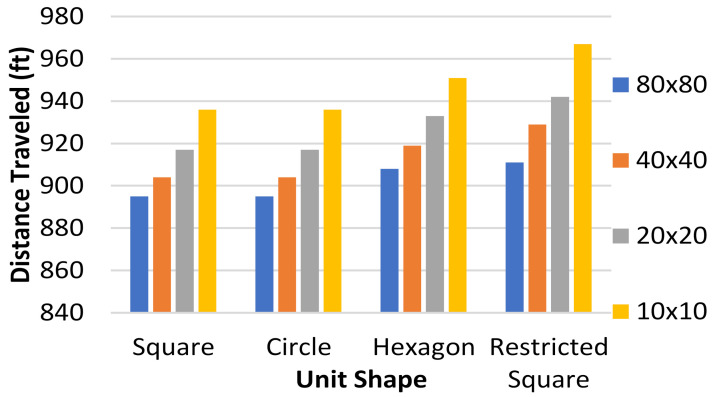
Comparison of the total distance traveled in feet vs. the shape of the fundamental discretization unit used for grid sizes between 10 units × 10 units and 80 units × 80 units using the data gathered from the prior tests.

Figure 14 is a summary of all of the tests up to this point including the unit shape, grid size, and total distance traveled. The results shown suggest that when the algorithm has less DoF in generating a path, the paths that are generated will have a greater total distance. Two factors that affect it are the number of total units on the grid and the number of directions that the algorithm can check from each discretization unit. The fewer total units or the fewer directions of potential movement, the more total distance each agent must travel. A further decrease in the degrees of freedom or the total number of grid units would most likely result in a larger total distance, and an increase in either of these variables would likely result in a smaller total distance.

### 4.6. Combined Effect of DoF and Occupancy Percentages

**Figure 15:**
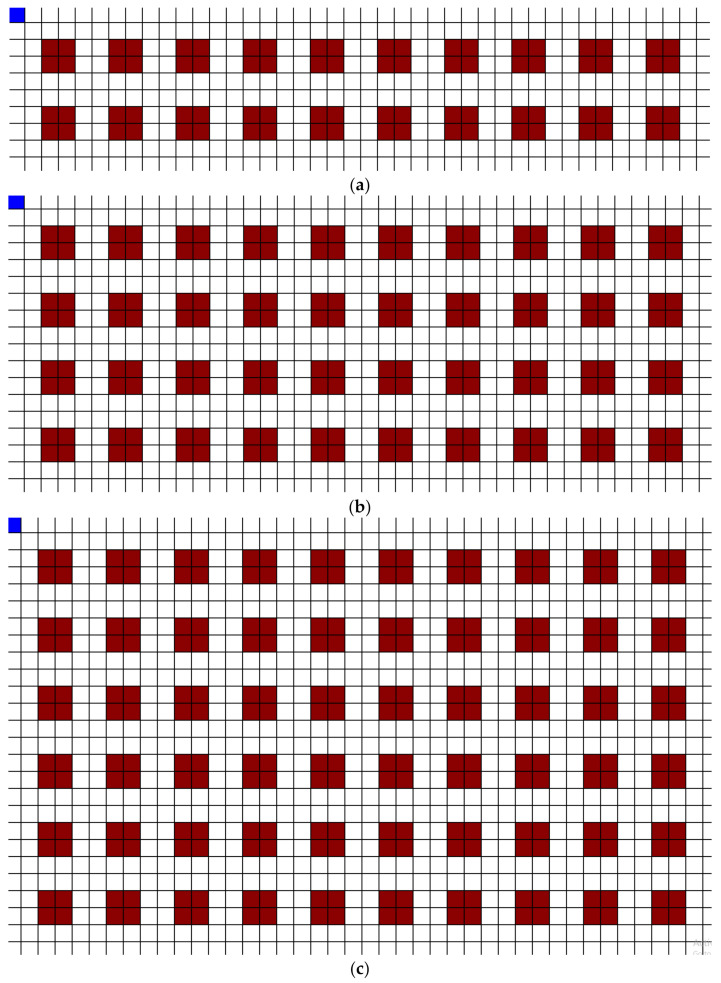
(**a**) 20 wards on a square-based grid (42 units × 10 units); (**b**) 40 wards on a square-based grid (42 units × 18 units); (**c**) 60 wards on a square-based grid (42 units × 26 units); each unit is 10′ × 10′.

In order to further test the effect of DoF using various occupancy percentages, the next tests tested various numbers of wards and occupancy percentages on a modified grid layout. This layout is shown in Figure 15a–c.

**Figure 16:**
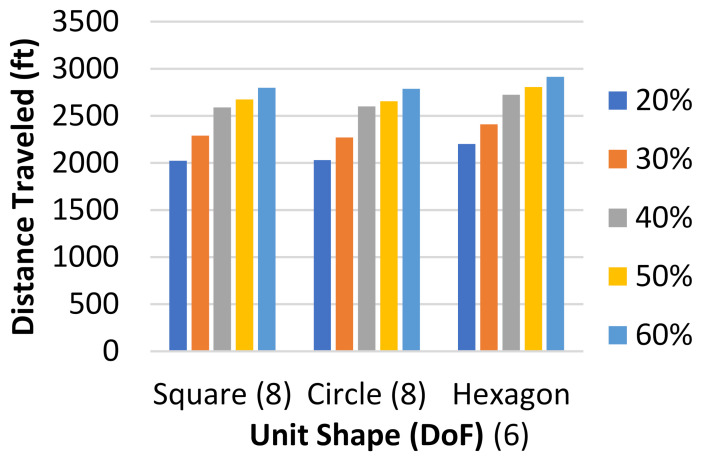
Total mean distance traveled in feet versus DoF for different occupancy percentages.

The occupied wards were randomly selected for each test. Each unit shape, which had differing DoF, was tested with 20, 40, and 60 total wards, and for each of these numbers of wards, 20%, 30%, 40%, 50%, and 60% occupancy were tested five separate times. For each of these tests, the total distance traveled by all three agents was measured and the results are displayed in Figure 16.

It should be noted that the circle and square results are nearly the same at all occupancy rates, which is to be expected because they both have the same DoF in the algorithm’s navigation. The hexagonal grid, however, only allows for six DoF instead of eight, and the consequences of this can be seen in the results. The increase in distance traveled was between 5% and 10% at all occupancy percentages when comparing the hexagon grid tests to the square and circle grid tests. The occupancy rates made no difference between different unit shapes, as through all unit shapes, the increase in distance stayed the same between occupancy percentages.

## 5. Safest and Mixed Paths

**Figure 17:**
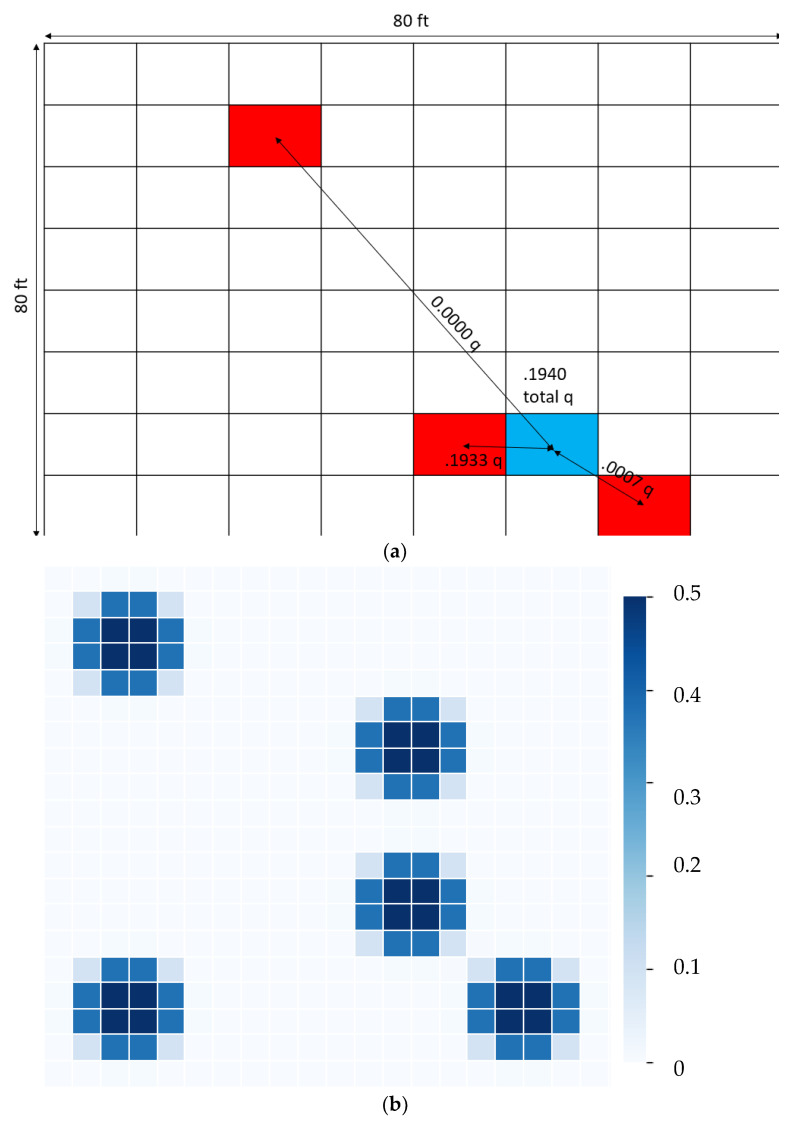
(**a**) Example of quantum being calculated for a given position on a grid. (Blue—potential point; red—infective patients; 3 infective patients, 8 units × 8 units grid) (**b**) Heatmap of quantum generated around 5 infective wards of a hospital (20 units × 20 units grid).

After running numerous tests on the validity of the algorithm used in this study and some of the controllable variables that may affect it, a dummy airborne infection model was introduced into the proposed framework. Several airborne infection models are available in the literature [26,27,28,29]. However, in this study, we employed a proxy exponential model to simulate an airborne infection model to simulate aggressive airborne diseases. This model was not created in this study but was, instead, applied to the tests conducted as a way to evaluate the amount of infection accrued by the HCP, also known as infection quantum or simply quantum. The quantum was the variable used in the algorithm to generate the ‘safest’ path through the MS hospital grid. The quantum was calculated for each step on a path using the equation given as:

(9) $$q=\beta e^{\left(\frac{-d^{2}}{2\sigma^{2}}\right)}$$

where β and σ are 50 and 3, respectively, and d is the distance between the susceptible person and the infective person in feet. Figure 17a provides an example of quantum being calculated at a given point. Figure 17b gives a heat map of quantum accrued for each position on a grid.

**Figure 18:**
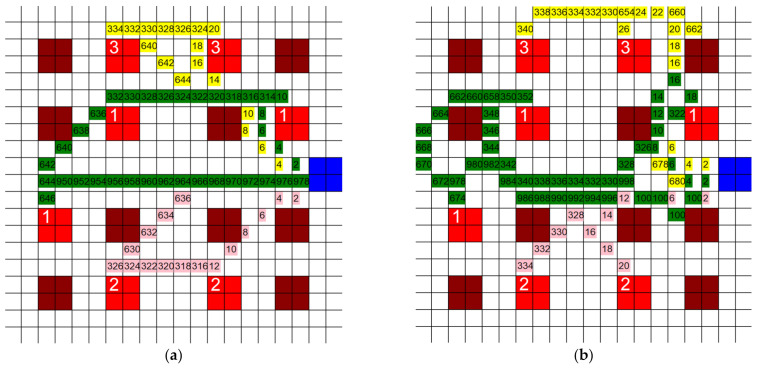

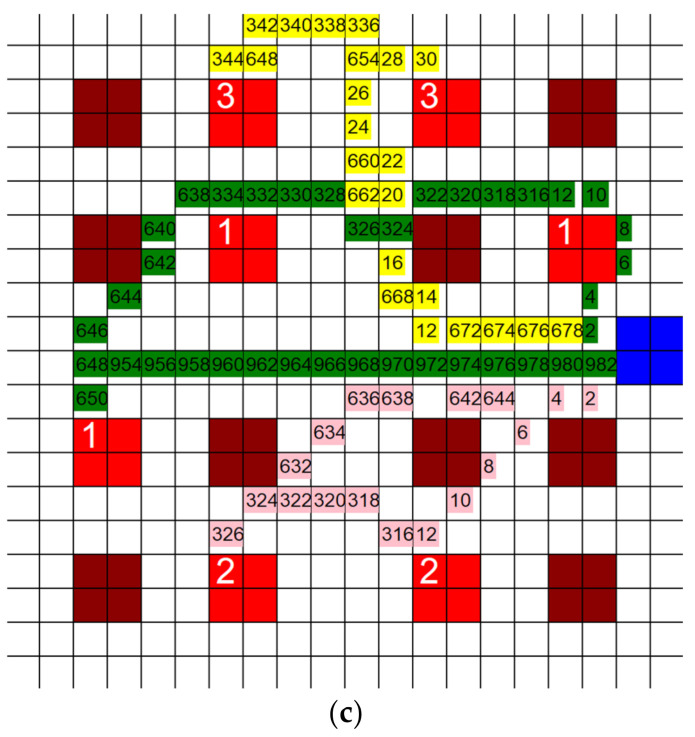
Example of (**a**) shortest possible path, (**b**) safest possible path, and (**c**) mixed path (7 infective patients, 20 units × 20 units, 3 HCP).

**Figure 19:**
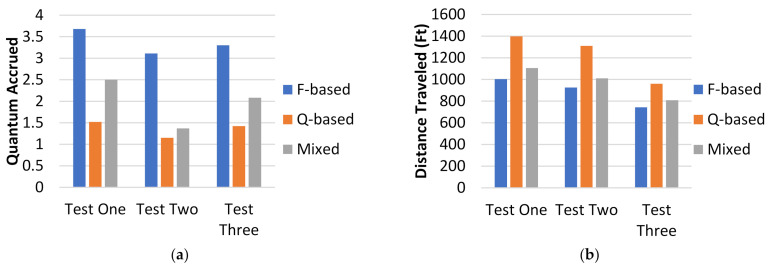

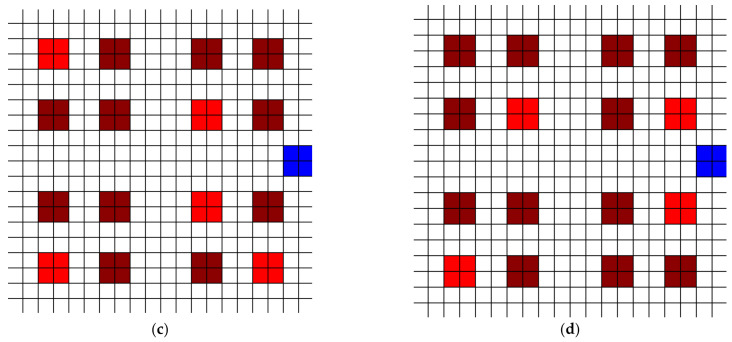
(**a**) Display of quantum generated for each test, (**b**) display of distance traveled for each test; (**c**) test two layout, (**d**) test three layout.

Three tests were ran using the layouts shown in Figure 18 and Figure 19c,d. For each grid, the position of the CR and wards remained the same. The only change made was the number and position of the infective patient rooms. For each test, three separate paths were generated for each agent using different criteria in each algorithm: one based purely on quantum, one based purely on distance, and the third being a mix of the two. All three modified algorithms used the same calculations for the F and quantum values $\left(q\right)$. An example of each of these three paths is shown in Figure 18.

The three paths generated are (1) the shortest possible path using the A* algorithm, also referred to as the *F*-based path, (2) the ‘safest’ possible path, also referred to as the q-based path, and (3) a combination of a short path and a safe path, also referred to as the mixed path. The *F*-based path is carried out using the same algorithm as all prior tests. Since this algorithm does not track the quantum generated, it will produce paths with more total quantum generated than the other two algorithms. However, it will also produce shorter paths than the other two algorithms. The path generated by this algorithm is demonstrated in Figure 18a.

The *q*-based algorithm is carried out entirely using the quantum value. It selects only the positions with the smallest quantum values and ignores the distance completely. This algorithm usually generates the path with the least amount of quantum generation, the safest path. However, it can be highly inconsistent as the lack in the tracking of distance results in very long paths such that the total quantum accrued can be less than the paths generated by the mixed path algorithm. The path generated by this algorithm is demonstrated in Figure 18b.

The mixed path algorithm is carried out by tracking both the distance and quantum at each position, hence the name ‘mixed’. First, all the positions with a smaller *F* to the current position are selected. Then, the quantum value of these positions is measured and the position with the smallest *q* is selected. This process assures that the algorithm is always moving towards the target position, while still making sure to accrue as little infection quantum as possible. This addresses the problem the q-based path algorithm faces. The path generated by this algorithm is demonstrated in Figure 18c.

Each path shown in Figure 18 has differences in its generation. This was one of the three tests ran. The results from Figure 18 are displayed as “Test One” in Figure 19a,b. For each test and each path in each test, the quantum generated by each agent was measured at each step and summed, and the total distance traveled by each agent was summed. The results for all the tests are shown in Figure 19a,b. The layouts of the other two tests are shown in Figure 19c,d.

From the graphs in Figure 19a,b, the quantum-based algorithm generates safer paths than the other two algorithms, but also generates significantly longer paths than the mixed path algorithm, and even more so when compared to the *F*-based algorithm. The q-based algorithm reduces quantum accrued by around 50–66% compared to the *F*-based algorithm, but the paths are also about 33–50% longer. When compared to the combination algorithm, the *F*-based algorithm generates paths with between 33% and 50% more quantum but only reduces the distance traveled by about 10–20%. Both the quantum-based and *F*-based algorithms generate paths with extreme distance and quantum values, respectively, but the combination algorithm consistently generates a quick path with relatively small quantum values.

**Figure 20:**
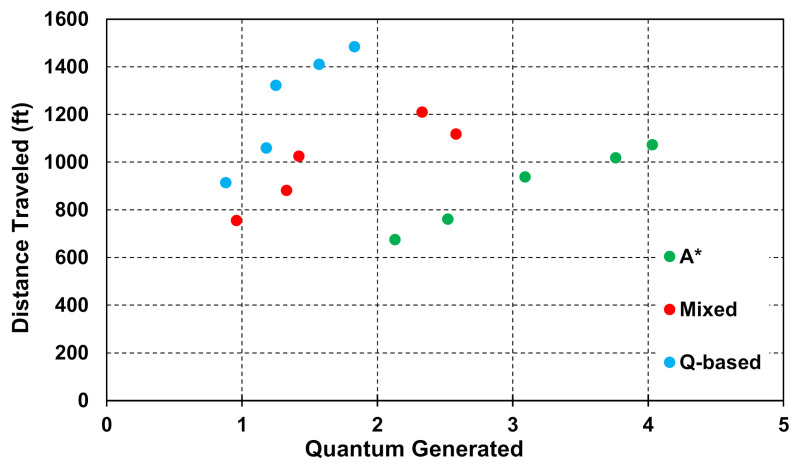
Graph of q-based (blue), F-based (green), and mixed path (red) algorithms’ results through five tests. Y-axis is total distance traveled by all three HCP in feet, and the X-axis is total quantum generated by all three HCP. Test number is denoted by the numbers above each point. (20 units × 20 units, 3 HCP).

Figure 20 shows a graph of total quantum generated and distance traveled through five tests for each of the three algorithms. Each algorithm is denoted by a separate color: the q-based is in blue, the F-based is in green, and the mixed path is in red. Each test is denoted by a number above the points; for example, all three of the points labeled “1” use the same layout of infective patients. All three tests use 3 HCP and the same overall grid size. It is clear to see that the q-based and mixed path algorithms are much more efficient at reducing quantum accrued by the HCP. However, the F-based algorithm still produces much shorter paths at the cost of doubling the quantum accrued in most tests.

In a “real world” scenario where algorithms such as these could be implemented, the HCP cannot afford to double their risk of infection (*F*-based algorithm) or travel a few hundred extra feet (quantum-based algorithm) every time they try to help patients. The combination algorithm would be the best option, despite it not creating the shortest or safest overall paths, due to its ability to mitigate both variables consistently.

## 6. Investigation of Infection Exposure Calculations

**Figure 21:**
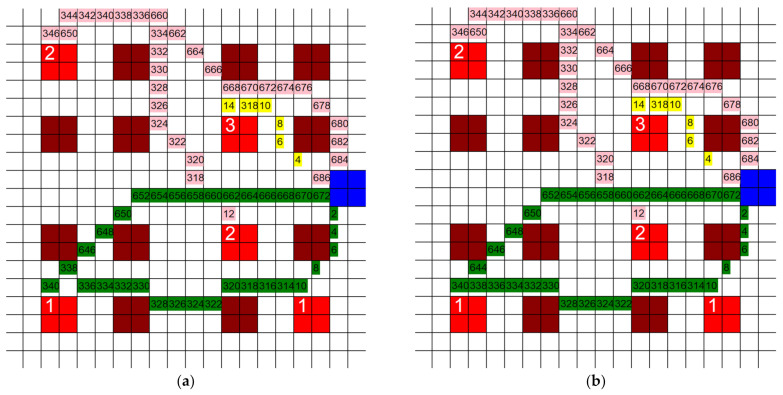
Example of a test run with the mixed path algorithm on a grid layout using different values for *β* and *σ*, (**a**) *β* = 50, *σ* = 3, and (**b**) *β* = 75 (3 HCP, 5 infective patients, 200′ × 200′ hospital layout).

**Figure 22:**
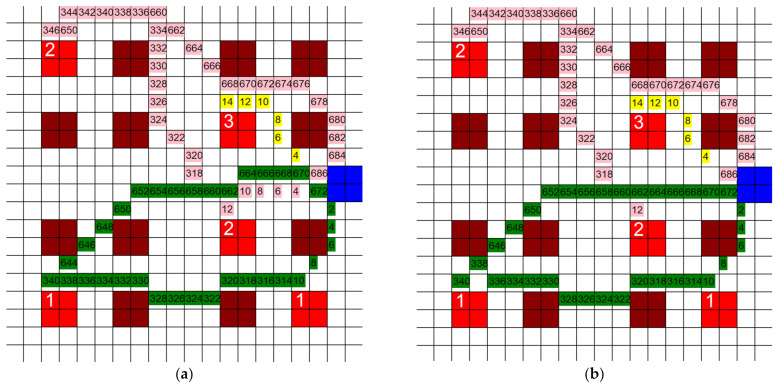

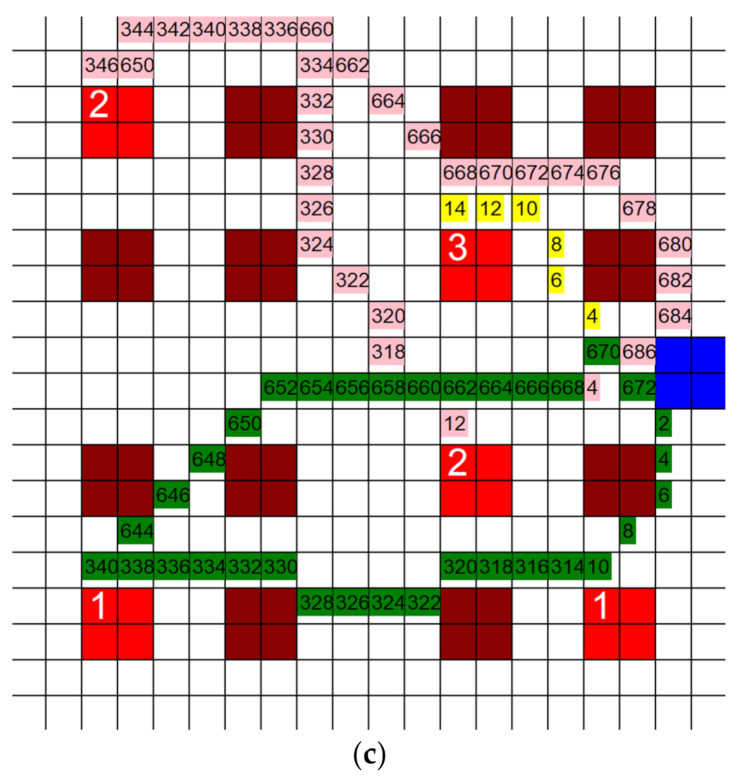
Example of a test run with the mixed path algorithm on a grid layout using different values for *β* and *σ*, (**a**) *β* = 200, *σ* = 7.5, (**b**) *β* = 25, *σ* = 1.5, (**c**) *β* = 150, *σ* = 6 (3 HCP, 5 infective patients, 200′ × 200′).

**Figure 23:**
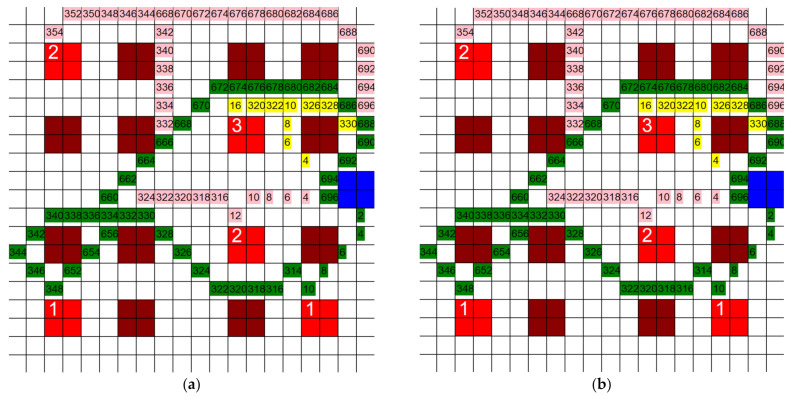
Example of a test run with the *q*-based algorithm on a grid layout using different values for *β* and *σ*, (**a**) *β* = 50, *σ* = 3, and (**b**) *β* = 75, *σ* = 4.5 (3 HCP, 5 infective patients, 200′ × 200′ hospital layout).

**Figure 24:**
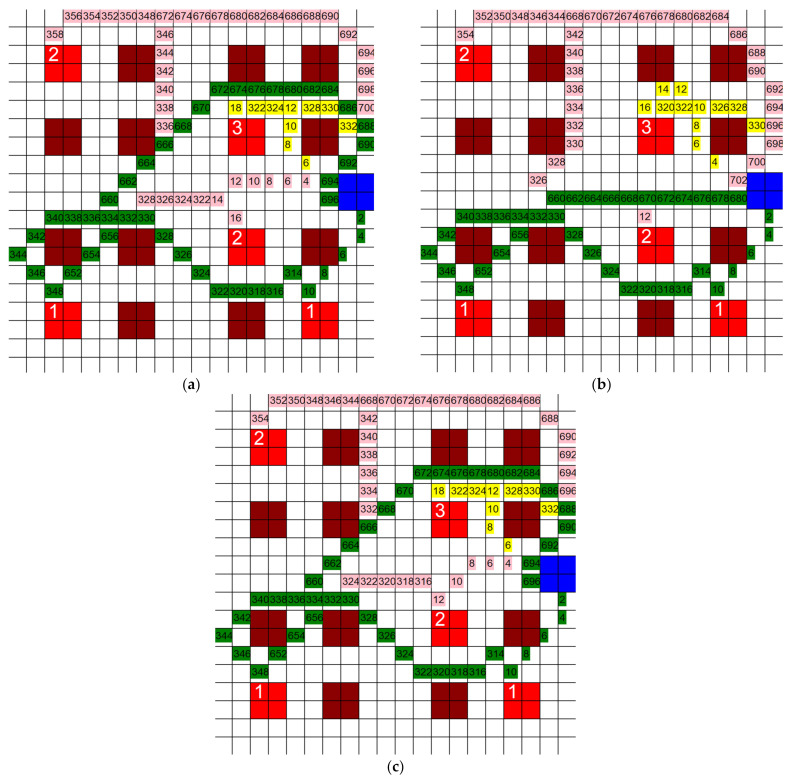
Example of a test run with the *q*-based algorithm on a grid layout using different values for *β* and *σ*, (**a**) *β* = 200, *σ* = 7.5, (**b**) *β* = 25, *σ* = 1.5, (**c**) *β* = 150, *σ* = 6 (3 HCP, 5 infective patients, 200′ × 200′ hospital layout).

The equation used to calculate quantum generated in the prior tests used *β* and *σ* as 50 and 3, respectively, and this generation of quantum can be seen in Figure 17b. To prove this study is not reliant on these numbers as is, the same layout used in Figure 19c was ran using the following $\left(\beta ,\sigma \right)$ sets: (25, 1.5), (75, 4.5), (150, 6), and (200, 7.5). Both the q-based and mixed path algorithms were tested using these sets of variables. The results for the mixed path algorithm are shown in Figure 21a,b and Figure 22a–c, and the *q*-based algorithm results are shown in Figure 23a,b and Figure 24a–c.

Looking at the grids in Figure 21 and Figure 22, there were only slight changes in distance between each of these algorithms, ranging from 1012′ to 1020′. The q values are different, but since the path generation is almost the same, it would not matter. It can be noted that there are slight differences between each of the graphs, but it would not be significant enough to impact the core function of the algorithm.

The grids in Figure 23 and Figure 24 use the same layout as the ones in Figure 21 and Figure 22. The only difference between the tests is the type of algorithm, either the *q*-based or mixed path algorithm, respectively. All of the grids in Figure 23 and Figure 24 are similar with a few minor changes each, excluding Figure 24b, in which the green path cuts straight across to the ward rather than looping around like in the others. Other than (b), the distances from the other four grids are all within 1322′ to 1356′. The distance from (b) is 1204′ and is an outlier from the rest of the data set. If this algorithm were to be implemented or expanded on, it would need to be noted that the values used in the calculation of the quantum generated might affect the outcome of the algorithm, as seen here.

## 7. Discussion

The work conducted in this study has some limitations that must be considered while examining the results. First, for new airborne diseases such as COVID-19, there is no full understanding of how to simulate its transmission. As more is learned about the disease, the results in this study could be expanded. Second, the equation used to calculate quantum generated is not specifically tailored to any specific disease including COVID-19, and is a generic estimation based on other airborne illnesses. Changing or modifying this equation could yield vastly different results than the ones found in this study. Third, the tests ran in this study are based on the idea that the agents can only have a set number of DoF, which works well when working with robots, but humans do not think in a set number of directions. Humans can move in any number of directions with different stride lengths and speeds. Changing either of these variables would directly affect the amount of quantum generated, which would affect the risk of the HCP contracting the disease. Finally, the number of agents used in this study would not be indicative of how many there are in a real MS hospital. The size of the MS hospital used in this study would only be a small fraction of the size of real MS hospitals and has only a tiny amount of HCP compared to real MS hospitals. Greatly increasing the size of the grid or the number of agents could affect the results found in this study.

The algorithms used in this study would be able to be implemented within real hospitals using a system at a workstation or desk on each floor of a hospital, assuming there is more than one. A floor layout of the hospital floor would need to be hashed up into a grid to be used. Workers could then hold phones, pagers, or any device with a screen and an ability to connect to the system. Something like augmented reality glasses, Google Glass for example, would also be usable, which could overlay the routes the workers needed to take as well as plenty of other important information about the patient. From there, workers would be able to see patients that need assistance, and the algorithm would be able to use their current position, as well as the position of other workers and infective patients, within the hospital floor to generate them a safe path to their desired destination. The algorithm can function in real time, and as soon as a patient would require assistance, a path can be generated for any available worker from their current position. It would function similar to a GPS, only on a much smaller scale and only when patients would require assistance would it generate paths.

The most common implementations of pathfinding algorithms, in GPS, video games, etc., use distance as the primary variable in the algorithm, which means they really only look for the shortest path(s). The algorithms used for the mixed and quantum-based paths in this study demonstrate pathfinding algorithms that either do not use distance or use multiple variables in determining the path. These algorithms that do not aim to create only the shortest path have many possible implementations. Navigation through a store is one example. Using a mock store layout and an expansion on the algorithms used in this study, there could be an algorithm developed that would create a path through a given grocery store that would help the user collect all the items they wanted. This is an example where there would need to be more than just the distance considered to assure that the user did not have to do lots of backtracking or cross the same areas more than once. This could then be expanded to also incorporate the infection reduction that was covered in this study. This algorithm could get the shoppers where they need to go both efficiently and safely by following one-way aisles and other COVID-19 spread-reducing measures implemented in superstores.

## 8. Conclusions

The important conclusions of this study are:

1. It was found in this study that an A* pathfinding algorithm can be used to generate the shortest path between two points on a grid of uniform squares, circles, and hexagons. The effect that the size of the fundamental discretization units had on the total distance traveled by all the agents was measured, and it was found that the smaller the units were, the less total distance the agents needed to travel to finish their paths. Conversely, when the unit size was increased, the total distance increased. The reasoning behind this is that when there are more units to choose from, slight optimizations are able to be made, such as cutting a corner shorter than usual. This was consistent across all shapes used as the fundamental discretization unit in this study.
2. The DoF of the algorithm was also tested and the effect it had on the total distance traveled was tracked. This was carried out by reducing the DoF and changing the shape of the fundamental discretization unit. Uniform circles, squares, and hexagons were all used. Uniform circles and squares both had eight DoF, hexagons had six, and squares were used again, but restricted to only travel in four directions instead of eight, meaning they had four DoF. The uniform circles and squares had the same results in all tests, which is expected since the DoF was maintained. The hexagons had a longer total distance than the uniform circles and squares, and the restricted square tests yielded even longer distances. These results are to be expected since the fewer options the algorithm has for each position, the longer the total distance traveled will need to be. The DoF was not increased from the 8 that were used to start because anything more than 8 is hard to produce on a graph due to the lack of uniform shapes that would allow it. Had the DoF been increased, it would be expected that the total distance traveled would be decreased.
3. Next, the DoF was tested again on different MS hospital sizes and occupancy percentages. Mock MS hospital layouts with 20, 40, and 60 total wards were all tested with occupancy percentages between 20% and 60%. The wards that contained patients were randomly selected for each test. Squares, circles, and hexagons were the fundamental discretization units used for these tests. Even with the different layouts and occupancy percentages, the results aligned with the prior DoF tests. The less DoF, the longer the paths generated were. The increase in total wards and occupancy percentages did not alter the expected results.
4. The next tests involved two separate algorithms that would aim to reduce the chance of infection rather than just find the shortest path. The q-based algorithm created the safest overall paths without consideration for the distance at all. Compared to the F-based, shortest path algorithm, it generated paths that were around 33–50% longer in the total distance. However, these paths also only generated about 33–50% as much infectious quantum, meaning that they were much safer, but took much more distance to travel.
5. The mixed path algorithm took consideration of both distance and quantum generated in order to mitigate both variables as much as possible. When compared to the F-based algorithm, the mixed path algorithm-generated paths were only about 10–20% longer but generated about 50–66% as much quantum. These paths are neither the safest nor fastest. However, they would be the most useful due to their lack of quantum generalization while still creating a path that is not hundreds of feet long.
6. The mixed path algorithm and q-based algorithm both provide distinct advantages over the A* pathfinding algorithm when used in a hospital setting. Although the A* algorithm provides the shortest path of the three, both of the other two algorithms keep the HCP much safer, up to 50% less exposure to infection quantum. Both the q-based and mixed path algorithms could be introduced into hospitals to help keep HCP safer from airborne illness while still getting them to their destination effectively.
7. The methods used in this study have several limitations, such as the relatively recent emergence of COVID-19, the quantum equation being based on a generic airborne illness rather than COVID-19 specifically, and the mock MS hospital used being much smaller than a real MS hospital. However, these limitations could be overcome using the algorithms introduced in this study or an expansion of them. As COVID-19 is studied more, the equations used to estimate its spread of exposure will become more well-defined and accurate, and the mock MS hospital could be expanded upon to better represent a real-world MS hospital.
